# Patients with Musculoskeletal Disorders Presenting to the Emergency Department: The COVID-19 Lesson

**DOI:** 10.3390/ijerph19105891

**Published:** 2022-05-12

**Authors:** Gianluigi Pasta, Alberto Polizzi, Salvatore Annunziata, Catherine Klersy, Lorenzo Fenech, Mohammad Reza Dermenaki Farahani, Matteo Ghiara, Alberto Castelli, Eugenio Jannelli, Federico Alberto Grassi, Mario Mosconi

**Affiliations:** 1 Department of Orthopaedics and Traumatology, IRCCS Fondazione Policlinico San Matteo, University of Pavia, 27100 Pavia, Italy; g.pasta@smatteo.pv.it (G.P.); salvatoreannunziata89@gmail.com (S.A.); mohammadreza.dermenakifarah01@universitadipavia.it (M.R.D.F.); m.ghiara@smatteo.pv.it (M.G.); a.castelli@smatteo.pv.it (A.C.); f.grassi@smatteo.pv.it (F.A.G.); mario.mosconi@unipv.it (M.M.); 2 Unit of Clinical Epidemiology and Biometry, IRCCS Fondazione Policlinico San Matteo Foundation, 27100 Pavia, Italy; c.klersy@smatteo.pv.it; 3 Centre for Research in Health and Social Care Management (CERGAS), SDA Bocconi, 20136 Milan, Italy; lorenzo.fenech@sdabocconi.it; 4 Multidisciplinary Department of Medico-Surgical and Dentistry Specialties, Luigi Vanvitelli, University of Campania, 80138 Naples, Italy; eugenio.jannelli@libero.it

**Keywords:** musculoskeletal disorders, emergency department, COVID-19, coronavirus outbreak

## Abstract

Background: Musculoskeletal disorders (MSKDs) are the most common class of complaints among patients presenting for care in the Emergency Department (ED). There is a non-urgent patient population with musculoskeletal complaints attending ED services that creates a burgeoning waiting list and contributes to overcrowding in Emergency Departments (EDs), which is a major concern worldwide. The recent (Coronavirus disease-19) COVID-19 pandemic is an unprecedented challenge that is revealing the structural and situational strengths and weaknesses of healthcare systems. Methods: This study retrospectively and prospectively assessed patients presenting to the Emergency Department before and after the COVID-19 outbreak (from 21 February 2019 to 3 May 2019 and from 21 February 2020 to 3 May 2020) with non-traumatic or low-severity musculoskeletal conditions to test the hypothesis that these patients should have access to care outside the ED and that the COVID-19 outbreak has changed patients’ care and health perception. Results: A total of 613 patients were identified, and 542 of them (87.56%) participated in a personalized survey. From this number, 81.73% of the total accesses took place in 2019, and only 18.27% of the accesses took place during the first outbreak and lockdown. More than 90% of patients admitted to the ED accessed care during the day shift in both periods. A total of 87.30% of patients presenting to the ED with a MSKD followed their general practitioner’s (GP) advice/referral in 2019, and 73.87% did so in 2020. The differences in the means of transport to the ED was statistically significant (*p*-value 0.002). Conclusions: The outbreak and lockdown period confirmed that there is an inappropriate use of the ED related to patients with MSKD. However, the ED appears to be the only available solution for these patients. New services and pathways are therefore needed to enhance MSKD management and reduce ED crowding. Additional observational studies shall be developed to confirm and compare our findings with those of various EDs. The main limit of the inferential part of the study is probably due to the small sample of patients in 2020.

## 1. Introduction

Overcrowding in the Emergency Department (ED) is a major concern in healthcare systems worldwide [1]. This situation is of increasingly major concern, especially in light of the aging population and the increases in the prevalence of chronic diseases. The resources available in the ED are finite, and hence, there is a need to prioritize patients on arrival to receive emergency medical care. Among the determinants of overcrowding, the literature identifies the lack of services in other parts of the acute care system [2]. Related to this, there is an inappropriate use of urgent department services [3]. The acuity of a presenting condition, based on a combination of initial complaints and provisional diagnosis, forms the basis for such prioritization [4]. 

Musculoskeletal disorders are the most common class of complaints among patients presenting for care in ED; for this reason, they constitute an obvious area of interest for researchers investigating ED overcrowding and appropriateness of visits [5,6]. 

Musculoskeletal disorders can range from acute high severity, such as limb-threatening or fracture-dislocations, to acute low-severity problems, such as the majority of contusions, strains, and sprains, to chronic low-severity musculoskeletal pain syndromes. Acuity refers to the time course of a disease or condition; severity describes the urgency of the disease or condition, and its potential to become life- or limb-threatening or to cause unnecessary pain and suffering [6].

Musculoskeletal pain (MSP) conditions, a cause of disability worldwide, especially if chronic and non-traumatic (NTMSKD), represent more than 25% of all ED visits that ED teams must evaluate and treat every day [7,8]. The non-urgent patient population with such musculoskeletal complaints attending the ED and orthopedic outpatients for care creates a burgeoning wait list that stretches the available finite ED resources [4]. 

Usually, patients presenting with NTMSKD after being assessed by the ED staff, would be treated medically for their condition and discharged and sometimes be referred to outpatient orthopedic clinics [4]. For example, in the USA, 48% of patient presenting with low back pain in the ED continued to take opioid medication for three months following their ED access [8]. 

On 11 March 2020, the World Health Organization (WHO) officially declared COVID-19 a pandemic [9]. The Coronavirus disease (COVID-19), a disease caused by the coronavirus SARS-CoV-2, is a global pandemic affecting over three million people and has vastly impacted healthcare systems worldwide. 

SARS-CoV-2 is transmitted by respiratory aerosols and presents clinically with fever, fatigue, myalgias, conjunctivitis, anosmia, dysgeusia, sore throat, nasal congestion, cough, dyspnea, nausea, vomiting, and/or diarrhea. In the most critical cases, symptoms can escalate into acute respiratory distress syndrome, accompanied by a runaway inflammatory cytokine response and multiorgan failure [10].

With the COVID-19 pandemic, our community faces its biggest challenge in modern history; the pandemic has challenged the infrastructure of the healthcare systems worldwide. At first glance, the COVID-19 pandemic and the field of orthopedic surgery appear complexly disparate. In fact, orthopedic practice has been markedly affected by the emergence of the COVID-19 outbreak to cope with the pandemic; substantial changes were introduced to surgical practice and education all over the world. The surgical field has been influenced as a result of the massive redirection of medical attention and priority towards caring for the COVID-19 infected patients [11].

Locally, the orthopedic practice has been markedly affected by the emergence of the COVID-19 outbreak. Patients requiring urgent or early orthopedic care are still treated as soon as possible, not differently from routine workflows; alternatively, elective, non-urgent procedures are postponed or cancelled [12]. 

This study retrospectively and prospectively assessed patients presenting to the Emergency Department with non-traumatic, low-severity musculoskeletal conditions, before and after COVID-19 outbreak, to test the hypothesis that these patients should have access to care outside the ED and that the COVID-19 outbreak has changed patients’ care and health perception. 

## 2. Materials and Methods

The San Matteo Hospital at Pavia is a Level 1 trauma center and a university-based hospital serving a geographic region with a population of about 700,000 people. The Orthopedic Emergency Room is part of the DEA (Department of Emergency and Acceptance), with a dedicated team that carries out its assistance activity on a daily basis. Therefore, there is always an orthopedic surgeon available in the ED from 8:30 am to 8:30 pm.

After receiving approval from our Institutional Review Board (protocol code 20210075145), we conducted a retrospective and prospective observational study. We retrospectively reviewed triage notes and letters of discharge for the patients discharged from the ED from 21 February 2019 to 3 May 2019 and from 21 February 2020 to 3 May 2020 with a diagnosis of non-traumatic, low-severity musculoskeletal conditions. All patients’ clinical files were reviewed in order to assess the pain severity, duration of pain and symptoms, previous medications, and requests for work medical certificate. Moreover, we focused on the role of the general practitioner as a connection point between the patient and the hospital. 

After the end of the lockdown related to Coronavirus outbreak, we surveyed eligible patients for 12 months by an interview-based survey. Inclusion criteria included: a diagnosis of non-traumatic, low-severity musculoskeletal conditions, 18 years of age or older, Italian speaking, and capable of giving informed consent. Written informed consent was obtained. No inducements were offered for participation in the study.

We administered, to the patients 18 years of age or older, a customized survey, which included questions regarding demographics; daily and working activity before, during and after COVID-19 outbreak; details about the presenting musculoskeletal condition and its acuity; questions addressing barriers to access the healthcare outside the ED; questions about the general practice, including the distance from home to the general practice office; the availability of the general practice to perform visits; the patient’s perception of his/her general practice ability to deal with musculoskeletal problems; details regarding prior evaluation and treatment of the presenting complaint, including specialty care; and the primary and secondary reasons the patients came to the ED that day.

### 2.1. Study Endpoints

We aimed to compare the presenting characteristics and modalities of access between the 2019 and 2020 cohorts and to further evaluate whether the proportion of appropriate access, defined as an access to the ED that required therapy and or hospitalization, differed between years, both overall and by predefined subgroups, such as sex, pain rating scale considering visual analogue scale (VAS), prognosis, and day or night shift at the orthopedic room (Table 1).

### 2.2. Statistical Analysis

For each yearly cohort (2019 and 2020), we described continuous variables with the median and 25th–75th percentile and compared them with the Mann–Whitney U test; we reported categorical variables as counts and percentages and compared them with the Fisher exact test.

We estimated the proportion of appropriate access with its 95% exact binomial confidence interval (CI). We fitted generalized regression models extended to the binomial family to compute the difference in appropriateness and 95% CI between the yearly cohorts. We performed predefined subgroup analyses for a series of patients and access characteristics. To verify the presence of effect modification by year, we computed the interaction of each variable and year.

We used Stata 17 (StataCorp, College Station, TX, USA) for all computations; a 2-sided *p*-value < 0.05 was considered statistically significant.

At the end of the screening process, we identified 707 patients. Therefore, we considered only the patients who had been assigned the color code green at the nursing triage, thus reaching a total of 619 patients. A further 6 patients younger than one year of age were subsequently excluded. Out of 613 patients, 501 accessed the ER in 2019 compared to 112 in 2020. 

Overall, based on our inclusion criteria, 397 accesses to the ER were considered appropriate in 2019 (79%, 95%CI 75–83) and 94 (84%, 95% CI 76–90) in 2020; out of the 397 accesses, 185 were female patients and 212 were male patients, while, in 2020, out of the 94 accesses, 42 were female and 52 males.

When considering the appropriateness-based sample, the data show how, in 2019, 79% reached the ER during the day shift and 67% during the night shift, while, in 2020, 84% visited the ER during the day and 87.5% at night.

When considering the prognosis, out of 501 accesses to the ER in 2019, 257 were given a prognosis of ≤7 days and 140 a prognosis of more than 7 days, while, in 2020, respectively, 56 were given a prognosis of ≤7 days and 49 a prognosis of more than 7 days.

In addition, the analysis of visual analogue scale categories (VAS) shows that the total number of patients in 2019 who reported a subjective VAS value lower or equal to 9 was 271, and the total number who reported a VAS value higher than 9 was 80, compared to 62 and 18 patients, respectively, in 2020.

When considering the sample according to the age of the patients, in 2019, 81% of patients were younger and equal to 49 years of age, while 77% of patients were older than 49 years of age. In 2020, the percentage of patients younger and equal to 49 years of age was 84%, while 84% of patients were older than 49 years of.

The number of patients who visited the ER during the week shift was 279 in 2019 and 60 in 2020, whereas the number of patients who visited the ER during the weekend shift reached a total of 118 in 2019 and 34 in 2020.

## 3. Results

At the end of the screening process, we identified 707 patients. Therefore, we considered only the patients who had been assigned the color code green at the nursing triage, thus reaching a total of 619 patients. 

An additional six patients younger than one year of age were subsequently excluded. The 2019 and 2020 cohort characteristics are shown in Table 2. The most striking differences regarded how the patients reached the hospital. 

The most striking differences concern how the patients reached the hospital; in 2019, out of 501 patients, 347 patients reached the hospital by their own vehicle, compared to 84 in 2020. In 2020, 62 patients arrived by advanced rescue vehicle versus 27 patients in 2019; 30 patients arrived by basic rescue vehicle in 2020 versus 9 patients in 2019, while the patients who arrived by emergency services numbered 58 in 2019 and 29 in 2020.

The percentage of the patients presenting to the ED following their GP’s advice/referral was three in 2019 and zero in 2020. 

In 2019, 87.3% (433 patients) of the ED visits were based on the patients’ decisions, compared to 73.9% (82 patients) in 2020.

**Figure A1:**
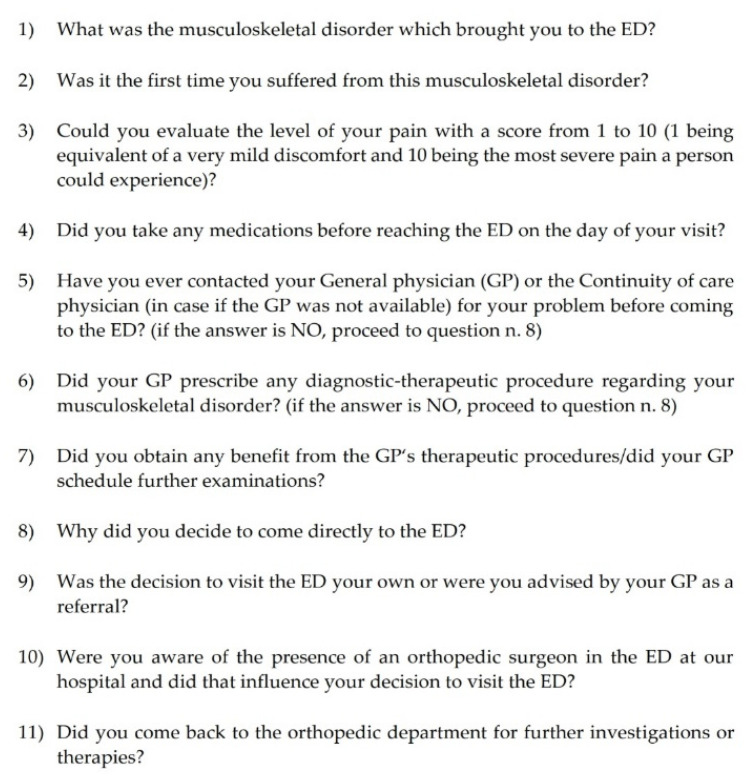
Evaluation survey of patients accessing the ED with a diagnosis of non-traumatic, low-severity musculoskeletal conditions.

Through the personalized survey (Figure A1) we developed, in which 542 patients participated, the subjective perception of pain through the VAS was investigated. Of the total number of patients, 417 had a VAS lower than or equal to nine. More specifically, in 2019 the percentage of patients was 77.08% versus 76.29% in 2020.

Overall, 397 accesses to the ER were considered appropriate in 2019 (79%, 95% CI 75–83) and 112 (84%, 95% CI 76–90) in 2020. The moderately higher rate during the pandemic (by 5%, 95% CI −3% to 12%), though suggestive of higher appropriateness, did not reach statistical significance (Table 1). Comparisons within the predefined subgroups are shown in Table 1. In all cases, we found neither a significant difference between years, nor the presence of interaction.

## 4. Discussion

ED overcrowding is a multifactorial problem and includes patient-related factors, such as aging population and increasing disease complexity, hospital-related factors, such as capacity, patient flow and ratio of ED to inpatient beds, and systems-related factors include access to primary care physicians [1]. 

Patients with musculoskeletal disorders represent a considerable percentage of ED volume. Although patients with acute or high-severity conditions are encouraged to seek care in the Emergency Department, patients with non-acute, low-severity conditions may be better served elsewhere. The American College of Emergency Physicians (ACEP) states that the ED should be used for “an unforeseen conditions which a prudent layperson, possessing an average knowledge of health and medicine, would judge to require urgent and unscheduled medical attention most likely available, after consideration of possible alternatives, in a hospital emergency department” [13]. However, a considerable percentage of ED visits by patients for musculoskeletal problems are non-acute, low-severity visits, and these patients are more likely to choose to come to the ED for evaluation at a time that is convenient for them. Our data, taken from the survey, suggest a large percentage of patients came to the ED for pain relief, to obtain a diagnosis, or to have access to radiography.

The COVID-19 pandemic has challenged the infrastructure of the healthcare systems worldwide. The orthopedic practice has been markedly affected by the emergence of the COVID-19 outbreak. In fact, to cope with the impact of the pandemic on healthcare systems, substantial changes were introduced to the surgical practices all over the world. The surgical field has been influenced as a result of the massive redirection of medical attention and priority towards caring for the COVID-19 infected patients. This situation is directly related to the overflowing of the intensive care units by the critically ill COVID-19 patients during the pandemic era and the shortage of resources in the public health system. Nevertheless, patients requiring urgent or early orthopedic care are still treated as soon as possible [13,14]. Alternatively, elective non-urgent procedures are postponed or cancelled. 

The aim of this study was to assess the differences between the patients presenting to the emergency department with musculoskeletal disorders before and during the COVID-19 outbreak. Principally, our study opted to analyze the changes in the delivery of emergency care throughout the first wave of COVID-19 outbreak. 

We observed that, in the time periods considered, the difference in the number of accesses is noticeable. In fact, 81.73% of the total accesses took place in 2019, and only 18.27% of the total accesses took place during the first lockdown. This pattern is in line with current literature findings.

In their theoretical model, Nadarajan G.D. et al. show that the pandemic generates three phases: pre-outbreak, outbreak, and post-outbreak. In the first two phases, non-COVID patients’ arrivals tend to decrease compared to normal activity, due to government restrictions (lockdowns) or fears of getting infected in hospitals [15]. In fact, this turns out to be the biggest limitation of this study. That is, the psychological implication of the pandemic caused a different perception of health status. There is indeed growing literature on the impact of pandemics on demand for EDs that confirms this pattern [16,17,18]. Casalino at al. analyzed admissions to four French hospitals and found that, during the lockdown period, there was a notable reduction in the number of non-COVID ED visits and admissions to medical/surgical wards and the intensive care unit (ICU) [16]. Garrafa et al. studied one of the most burdened hospitals, hit by the first pandemic wave in Italy, and found that there was a dramatic decrease in access to the ED beginning just after the announcement of the lockdown and continuing throughout the most acute phase of the pandemic. The reduction was mostly related to less urgent non-COVID codes (white or green) but also to urgent (yellow and red) codes experienced some reduction [18]. Castoldi et al. showed that, immediately before and during the COVID-19 lockdown, there was a significant decrease in the overall volume of EDs’ attendances in Lombardy, compared to the same time interval in 2019 [17]. Alternatively, San Matteo hospital is the provincial reference center for trauma management and for non-traumatic musculoskeletal conditions due to the fact that there is direct access to the musculoskeletal pathway in the ED. Moreover, during the pandemic lockdown, the other hospitals in the province of Pavia were readdressed to increase the number of beds for the management of COVID-19 patients; despite this, our institution continued to visit and treat non-traumatic musculoskeletal conditions with a dedicated pathway. 

As for orthopedic surgeries, it is possible that the reduction was related to orthopedic traumas that were associated with home confinement and the drastic reductions in vehicle traffic. The pandemic is an unprecedented event with an unprecedent impact on ED demand. Inappropriate acute NTMSKD demand is a peculiar, as well as relevant, variable of ED crowding that needs to be addressed. This study focuses on the impact of the pandemic on NTMSKD demand and admissions to the ED in order to draw lessons for future pandemics as well as returning to “business as usual” scenarios.

The predefined subgroup analysis showed that there is no statistically significant difference in access during the day and night shifts in the two time periods considered. In both 2019 and 2020, more than 90% of patients admitted to the ED accessed it during the day shift. The reason behind this phenomenon might be that when patients weigh the value of each of the influences, such as desire for pain relief, obtaining a diagnosis, or the possibility to get radiographs, they make the decision to come to the ED for evaluation and treatment instead of visiting their general physicians. The fact that these patients were more likely to come to the ED during regular office hours and less likely to come during evenings and on weekends suggests that the ED is used as a place of convenience and that these patients are being seen when it is easiest for them to allot the time according to the priorities of their daily lives. This is supported by the ED usage patterns, related to time of the day and day of the week, seen in patients presenting to the ED with non-acute, low-severity musculoskeletal problems. When the problem is of a non-acute, low-severity nature, patients are more likely to wait for a more convenient time to come to the ED (Monday through Friday, 8:30 a.m. to 8:30 p.m.) [3]. 

Concerning the eventual hospitalization of the patients reaching the ED who sought medical attention during the COVID-19 outbreak, our data suggest an important increase in the number of patients transferred to the inpatient department for further treatments in 2020. During the trimester period from 21 February to May 2020, 7.14% of the total patients were hospitalized from ED in the Traumatology Department in comparison to 3.59% of the total in the same trimester in 2019. These data suggest encounters with more serious conditions during the first wave of the COVID-19 outbreak in comparison to the same trimester in 2019.

Furthermore, we observed slightly more serious conditions in terms of the duration of prognosis during the COVID-19 outbreak in comparison to the same trimester in 2019. Our study observed that, during the trimester period from 21 February to May 2020, from the total number of the patients who visited our ED with MSKD, 43.75% at discharge received a prognosis of longer than seven days for their conditions. Alternatively, during the same trimester in 2019, 34.13% of the patients who visited our ED with MSKD received a prognosis longer than seven days.

Concerning the means of transport of the patients with MSKD visiting our ED, we observed a slight increase in terms of the number of patients who reached the ED by ambulance. Here, 14.68% of the total accesses took place during the first wave of the COVID-19 outbreak, in comparison with the same period in 2019, in which only 12.38% of the total number of the patients with MSKD used an ambulance to reach the ED. Moreover, during the first wave of the COVID-19 outbreak, we recorded a modest reduction in the number of autonomously transported patients and, consequently, an increase in those transported to the ED by the territorial emergency service. 

Our study further demonstrates that the percentage of the patients presenting to the ED with MSKD following their GP’s advice/referral during the COVID-19 established lockdown was subjected to a 13.43% decrease in comparison to the same time period in 2019 (87.30% in 2019 compared to 73.87% in 2020). Despite this, a large percentage of patients still present to the ED following a GP’s advice/referral. 

Regarding the pain assessment in our study, we employed the numeric rating scale VAS (from 1 to 10), which is a commonly used scale, mainly because of its easy application. We are aware that pain is a subjective feeling, and the self-assessment of pain by the patient can be influenced by a variety of factors, including, but not limited to, socio-economic status, beliefs, and psychological status. Based on our observations, during the first wave of the COVID-19 outbreak, even the perception of pain tolerance was subjected to changes to a certain degree. Of the total number of patients, 417 had a VAS lower than or equal to nine; in 2019, the percentage of patients was 77.08% in comparison with 76.29% in 2020. Therefore, this result might suggest that during the pandemic lockdown, a slightly higher level of pain was tolerated by patients before bringing them to visit the ED. This difference could be the result of a variety of factors; however, we presume that the main cause of the tendency to tolerate a higher pain threshold may be due to the fear of risking infection by the COVID-19 virus in the hospital setting and, specifically, at the ED.

## 5. Conclusions

The study shows that there is an inappropriate use of the ED related to patients with MSKD. However, the ED appears to be the only available solution for these patients. New care services and pathways, including home-based services, are therefore needed to enhance MSKD management and avoid ED crowding. Shifting patients from the ED to outpatient GP offices and subspecialty practices will require large-scale changes in attitude and restructuring of the currently used models of healthcare delivery in the Italian healthcare national system. 

The call emerges to construct a territorial-based program for an approach that enhances all the skills in the field, clearly aiming at a close collaboration between the hospital care system and primary care measures. Appropriate use of the emergency department by patients with musculoskeletal disorders may require not only increased access to primary care, but also improved public understanding of the scope of care offered by primary care physicians and the conflicting demands placed on Emergency Department providers. For example, national guidance and awareness campaigns have to be developed on best practices for the treatment of common chronic conditions in primary care. 

We are aware that this study is limited by not being a multicenter study and that it was developed in a university hospital. These aspects certainly affected the variable number of admissions to the ED, but the paper highlights results that need to be carefully considered and further studied. This is why additional observational studies are needed to see if our findings hold true at various EDs. Prospective studies are needed to determine whether patients with non-acute, low-severity musculoskeletal problems can be safely routed to other venues of care. 

## Figures and Tables

**Table 1:** Appropriateness of access in the ER by year, overall, and by predefined subgroups.

Subgroup	Year	N	Appropriateness N (%)	Difference (95% CI)	*p*-Value	*p* for Interaction with Year
-	2019 2020	501 112	397 (79%) 94 (84%)	Ref 5% (−3 to 12)	0.296	-
Sex Female Male	2019 2020 2019 2020	234 48 267 64	185 (79%) 42 (87%) 212 (79%) 52 (81%)	Ref 8.4% (−2.2 to 19.1) Ref 1.8% (−8.9 to 12.5)	0.263 0.863	0.394
Shift Day shift Night shift	2019 2020 2019 2020	498 104 3 8	395 (79%) 87 (84%) 2 (67%) 7 (87.5%)	Ref 4.3% (−3.6 to 12.3) Ref 2.1% (−3.7 to 7.9)	0.347 0.491	0.581
Prognosis ≤7 >7	2019 2020 2019 2020	330 56 171 49	257 (78%) 56 (89%) 140 (82%) 38 (76%)	Ref 11% (2.1 to 20) Ref −4.3% (−17.3 to 8.7)	0.059 0.538	0.057
VAS ≤9 >9	2019 2020 2019 2020	343 74 102 18	271 (79%) 62 (84%) 80 (78%) 18 (78%)	Ref 4.8% (−4.7 to 14.2) Ref −0.2% (−18.8 to 18.5)	0.425 0.437	0.643
Age ≤49 >49	2019 2020 2019 2020	251 56 250 56	204 (81%) 47 (84%) 193 (77%) 47 (84%)	Ref 2.7% (−8.1 to 13.4) Ref −6.7% (−4.2 to 17.7)	0.402 0.368	0.603
Shift Week shift Weekend shift	2019 2020 2019 2020	353 74 148 38	279 (79%) 60 (81%) 118 (80%) 34 (89%)	Ref 2% (−7.8 to 11.9) Ref 9.7% (−2 to 2.1)	0.754 0.239	0.325

**Table 2:** Description of patients by yearly cohort; data are reported as N (%) unless otherwise specified.

Variable	Year 2019 (N = 501)	Year 2020 (N = 112)	*p*-Value
Age [years] (median, 25th–75th)	49 (34–64)	49 (36–62)	0.521
VAS (median, 25th–75th)	9 (8–9)	9 (8–9)	0.492
Male	267 (53)	64 (57)	0.465
Day shift	3 (0.60)	8 (7.14)	0.000
Week shift	353 (70.46)	74 (66.07)	0.365
Therapy	108 (21.56)	19 (16.96)	0.305
Hospitalized	18 (3.59)	8 (7.14)	0.116
Prognosis ≤ 7 days (median, 25th–75th)	330 (5–10)	63 (5–10)	0.077
Own vehicle	437 (87.23)	84 (75)	0.003
Advanced rescue vehicle	62 (12.38)	27 (25)	0.003
Basic rescue vehicle	30 (5.99)	9 (8.04)	0.003
Law enforcement	2 (0.4)	0 (0)	0.003
Hospital vehicle	2 (0.4)	0 (0)	0.003
By themselves	433 (87.3)	82 (73.9)	0.002
From another hospital	1 (0.2)	0 (0)	0.002
Emergency services	58 (11.7)	29 (26.1)	0.002
General practitioner	3 (0.6)	0 (0)	0.002
Emergency medical station	1 (0.2)	0 (0)	0.002
